# Letter from Vienna—Cæsarean Section

**Published:** 1879-11-20

**Authors:** 


### *LETTER FROM VIENNA—CÆSAREAN SECTION.*

MR. EDITOR:—On Sunday, May 25th, I had the good fortune to see a case of Cæsarean section with extirpation of the uterus and both ovaries, a description of which may be of interest to your readers. This operation, which originated in America, has lately been revived here, and is now well established, having been done, in all, twenty-two times, and seven times in Vienna alone. Professor Carl Braun has operated three times previous to the operation I am about to describe. One of the patients was in a very bad condition at the time of the operation, and died soon after, but the other two cases were successful. Professor Spaeth has operated twice. In one case the patient was almost dead at the time of the operation, dying soon after, and a putrid child was extracted. In the second case both mother and child were saved. Professor Gustav Braun has operated once, the mother dying, and the child being saved.

Professor Carl Braun performed his fourth operation at 10:40 p. m., May 25th, in the lecture room, about fifteen spectators being present. The patient is a dwarf, four feet in height, and is twenty-five years old. She had rachitis when a child, and is frightfully deformed. She was raped by a drunken man, thirty-six years old, last August, and had no difficulty during her pregnancy, coming to the hospital soon after labor pains began, and being in apparently excellent condition, with the exception of a slight attack ot bronchitis. The abdomen was very large, the child being apparently of full size. The head presented, but was freely movable above the brim of the pelvis. The pelvis was of the rachitic type, with an antero-posterior diameter of two inches. The operation was performed soon after the beginning of labor, by Professor Carl Braun, assisted by Professor Gustav Braun and other gentlemen.

The patient was narcotized with a mixture of ether and chloroform, which is in general use here, the abdomen washed with carbolic acid and water, the pubes shaved, and the catheter introduced. The membranes had ruptured spontaneously about half an hour previously. An atomizer with a solution of thymol stood in the room, but the stream was not directed over the abdomen. The incision was made from the umbilicus downward within three inches of the symphysis, in the linea alba, and carefully deepened until the peritoneal cavity was opened. The arteries, two small branches, were secured by torsion, and then a probe-pointed bistoury was introduced, and the incision prolonged upward and to the left one and a half inches. The uterus was thus exposed, and was pushed forward by an assistant, so that its anterior surface protruded through the abdominal wound, and an incision being made with a scalpel, a probe-pointed bistoury was introduced, and the incision prolonged to about four inches. The gush of blood which followed was prevented from entering the abdominal cavity by the forward position of the uterus. The child was then extracted by the feet, and the placenta was torn off at the same time. The uterus was then grasped around the vaginal portion and compressed, the bleeding being controlled in this way until the chain of Billroth's ecraseur was adjusted. This was so applied as to inclose the uterus at the anatomical internal os, both ovaries thus being above the chain, and was strongly compressed. The uterus was then excised three-quarters of an inch above the chain, the ovaries being included in the excised mass. The stump of the uterus was then inclosed in a steel clamp, below the chain of the ecraseur, and, the latter being removed, the stump above the clamp was transfixed with a long needle, with the idea of preventing the clamp from slipping when sloughing began. Two rubber drainage tubes were then inserted, and the edges of the abdominal wounds were brought together, the stump and clamp remaining outside. Silk sutures were used, being inserted over a flat sponge, and tied after the sponge was removed. The wound was dressed with a Lister's bandage, some preparation of tar being applied immediately over the wound. The whole operation took just an hour, and the patient rallied well, and seemed much comforted at the promise of a glass of brandy. The child was a large girl, and was in excellent condition.

The whole operation was exceedingly well done, and four days later the patient was doing well, and seemed in a fair way to recover, though the bronchitis caused some anxiety. The advantages of this operation are, first, that the patient is never exposed again to the danger of a similar operation, should she survive; secondly, that the bleeding is absolutely controlled after the extraction of the contents of the uterus; and, thirdly, that the danger of peritonitis is much lessened by avoiding uterine sutures, and secondary hemorrhage from the uterine wound, which was so often the case when uterine sutures were not employed.

The results of the Vienna cases are certainly very favorable, and so far seem to recommend a wider adoption of the operation.—*Boston Medical Journal.*

